# Traditional Chinese Medicine Tanreqing Inhibits Quorum Sensing Systems in *Pseudomonas aeruginosa*

**DOI:** 10.3389/fmicb.2020.517462

**Published:** 2020-12-17

**Authors:** Weifeng Yang, Qing Wei, Qian Tong, Kaiyu Cui, Gaiying He, Longfei Lin, Lvyan Z. Ma, Pierre Cornelis, Yi Wang

**Affiliations:** ^1^Experimental Research Center, China Academy of Chinese Medical Sciences, Beijing, China; ^2^Department of Food and Environmental Sciences, University of Helsinki, Helsinki, Finland; ^3^School of Biological Engineering and Food Science, Hubei University of Technology, Wuhan, China; ^4^Institute of Chinese Materia Medica, China Academy of Chinese Medical Sciences, Beijing, China; ^5^State Key Laboratory of Microbial Resources, Institute of Microbiology, Chinese Academy of Sciences, Beijing, China; ^6^Department of Bioengineering Sciences, Microbiology Unit, Vrije Universiteit Brussel, Brussels, Belgium; ^7^Université de Rouen Normandie, Normandie Université, Laboratoire de Microbiologie Signaux et Microenvironnement, LMSM EA4312, Évreux, France

**Keywords:** *Pseudomonas aeruginosa*, antimicrobial agent, quorum sensing, two-component system, Tanreqing

## Abstract

*Pseudomonas aeruginosa* is an opportunistic pathogen that can infect a wide variety of hosts including humans, plants, and animals. The production of virulence factors is the determinant of the infection paradigm and is under orchestrated regulation via cell-to-cell communication process called quorum sensing (QS). To disable QS circuits and prevent bacterial infections, a large battery of anti-QS agents, particularly from traditional Chinese medicine have been developed. Here, we used *P. aeruginosa* as a model microorganism to investigate the effect of traditional Chinese medicine Tanreqing (TRQ) formula on bacterial pathogenicity. Phenotypic analysis showed that TRQ treatment could completely inhibit the production of phenazine pyocyanin and moderately inhibit the production of virulence factors such as rhamnolipids, elastase, and alkaline protease. Further transcriptomic analyses revealed that TRQ treatment could significantly attenuate the expression of QS-regulated genes in *P. aeruginosa* and TRQ-treated *P. aeruginosa* regulon shared a large overlap with QS regulon. Component contribution to QS inhibition shed light on the indispensable role of all five components in TRQ formula. Further genetic analysis indicated that upstream regulators of QS systems, including two-component systems GacS/GacA and PprA/PprB, were both inhibited by TRQ treatment. Finally, our TRQ formula could efficiently protect *Caenorhabditis elegans* from killing by *P. aeruginosa*. Altogether, we have proved TRQ formula as an effective and specific agent to attenuate bacterial virulence and combat bacterial infections.

## Introduction

*Pseudomonas aeruginosa* is an important human opportunistic pathogen that causes a panel of acute infections such as urinary tract infections, acute ulcerative keratitis and burn wound infections as well as pulmonary infections, as often seen in cystic fibrosis patients (Lyczak et al., 2000, 2002; Haussler, 2004). This bacterium is noted for its production of a large number of extracellular virulence factors including the phenazine pigment pyocyanin, lectins, siderophores, proteases and rhamnolipids that are required to initiate a successful acute infection (Jimenez et al., 2012). To efficiently coordinate these virulence gene expression, *P. aeruginosa* employs a ubiquitous bacterial cell-to-cell communication process called quorum sensing (QS) to produce, secrete, and detect the chemical signaling molecules termed autoinducers in response to fluctuations in cell population density (Camilli and Bassler, 2006; Papenfort and Bassler, 2016).

Two canonical LuxI/LuxR QS circuits (LasI/LasR and RhlI/RhlR) exist in *P. aeruginosa* (Schuster and Greenberg, 2006; Venturi, 2006). Both LasI and RhlI are autoinducer synthases that catalyze the formation of signal molecule *N*-3-(oxododecanoyl)-homoserine lactone (3-oxo-C12-HSL) and *N*-butyryl homoserine lactone (C4-HSL), respectively. LasR and RhlR function both to bind their respective autoinducers and to activate the transcription of downstream genes (Miller and Bassler, 2001). The two regulatory circuits act in tandem to control the expression of a large number of *P. aeruginosa* virulence factors (Parsek and Greenberg, 2000; Papenfort and Bassler, 2016).

In addition to classical autoinducers, *P. aeruginosa* produces quinolone signals including 2-heptyl-4-quinolone (HHQ) and 2-heptyl-3-hydroxy-4-quinolone (PQS) to regulate virulence gene expression (Diggle et al., 2006, 2007; Heeb et al., 2011). Both HHQ and PQS regulate gene expression via acting as the ligand of the MvfR regulator, also known as PqsR (Deziel et al., 2005; Xiao et al., 2006; Diggle et al., 2007). Although a fourth QS signal molecule, termed IQS, has been suggested (Lee et al., 2013), it later appeared that IQS is in fact aeruginaldehyde, a by-product of the siderophore pyochelin biosynthesis pathway and not of the AmbABCDE as proposed previously (Ye et al., 2014; Rojas Murcia et al., 2015; Cornelis, 2019). A yet uncharacterized autoinducer produced by PqsE that interacts with RhlR has been recently reported (Mukherjee et al., 2018).

In *P. aeruginosa*, QS systems not only act as central regulatory circuits for the expression of virulence factors, but also are involved in the development of biofilms, sessile communities of bacterial cells and the causative agent of chronic infections (Davies et al., 1998). Therefore, QS systems represent ideal and attractive targets for the design of novel therapeutics (Rasmussen and Givskov, 2006). Extensive studies have been undertaken to search for novel anti-QS drugs that could override bacterial communication signals and investigate their potential roles in the attenuation of virulence in *P. aeruginosa* (Hentzer et al., 2003; Muh et al., 2006; Borlee et al., 2010; Tan et al., 2013; Starkey et al., 2014).

Up to now, almost all *P. aeruginosa* QS systems have corresponding anti-QS drugs that could reduce the virulence in *in intro* and *in vivo* infection models (Fan et al., 2013; O’Loughlin et al., 2013; Starkey et al., 2014; Li et al., 2018). For example, azithromycin, a macrolide antibiotic, has been shown to inhibit the QS system in *P. aeruginosa* through several mechanisms (Nalca et al., 2006; Skindersoe et al., 2008; Perez-Martinez and Haas, 2011). However, the development of resistance to these anti-QS compounds and concerns over the safety of synthetic chemicals have been viewed as serious side effects of these types of compounds (D’Costa et al., 2011; Wright, 2011). To resolve these global health threats and determine effective anti-QS compounds, the use of natural products, particularly traditional Chinese medicine (TCM), has become attractive due to their broad spectrum of secondary metabolites and low potential to develop resistance (Koh et al., 2013; Asfour, 2018; Chong et al., 2018).

The Tanreqing (TRQ) injection is a Chinese herbal preparation made from *Scutellariae radix* (Huang Qin in Chinese, HQ), *Lonicerae flos* (Jin Yin Hua in Chinese, JYH), *Forsythiae fructus* (Lian Qiao in Chinese, LQ), *Ursi fel* (Xiong Dan in Chinese, XD) and *Naemorhedi cornu* (Shan Yang Jiao in Chinese, SYJ). According to TCM theory, TRQ formula could clear heat, detoxify and remove phlegm and has been used in China as a treatment for respiratory tract infections, pneumonia and chronic obstructive pulmonary disease (Liu et al., 2016). However, the mode of action of TRQ is still not well understood in respect to its antibacterial activity.

In this study, we used *P. aeruginosa* as a model microorganism to investigate the inhibitory mechanism of TRQ against bacterial infections. We found that TRQ formula could completely inhibit the expression of the three QS systems in *P. aeruginosa* at sub-minimum inhibitory concentrations (sub-MIC) *in vitro* and *in vivo*, suggesting that TRQ is a highly specific and effective agent to attenuate bacterial virulence and combat bacterial infections.

## Materials and Methods

### Bacterial Strains and Culture Conditions

Bacterial strains and plasmids used in this study are shown in Table 1. Both *P. aeruginosa* and *E. coli* strains were grown in Lysogeny broth (LB) with aeration at 37°C. When required, LB agar plates were used to streak bacterial colonies. In addition, antibiotics were added into the growth medium at the following concentrations: 300 μg ml^–1^ carbenicillin (Cb) for *P. aeruginosa*, 100 μg ml^–1^ ampicillin (Amp) for *E. coli*.

**TABLE 1:** Strains and plasmids used in this study.

Strain/plasmid	Characteristics	References
***Pseudomonas aeruginosa***		
PAO1	Wild-type; ATCC 15692	Wei et al. (2011)
PAO1/pProbe-AT′	PAO1 containing promoterless *gfp* reporter plasmid pProbe-AT′, Cb^*r*^	This study
PAO1/plasR-gfp	PAO1 containing reporter plasmid pProbe-lasR, Cb^*r*^	This study
PAO1/pmvfR-gfp	PAO1 containing reporter plasmid pProbe-mvfR, Cb^*r*^	This study
PAO1/prhlR-gfp	PAO1 containing reporter plasmid pProbe-rhlR, Cb^*r*^	This study
PAO1/pgacS-gfp	PAO1 containing reporter plasmid pProbe-gacS, Cb^*r*^	This study
PAO1/pgacA-gfp	PAO1 containing reporter plasmid pProbe-gacA, Cb^*r*^	This study
PAO1/pcyaB-gfp	PAO1 containing reporter plasmid pProbe-cyaB, Cb^*r*^	This study
PAO1/pcupE1-gfp	PAO1 containing reporter plasmid p*cupE1*-PUCPgfps, Cb^*r*^	This study
PAO1/ppprB-gfp	PAO1 containing reporter plasmid p*pprB*-PUCPgfps, Cb^*r*^	This study
PAO1/prpoS-gfp	PAO1 containing reporter plasmid pProbe-rpoS, Cb^*r*^	This study
PAO1/pdgcH-gfp	PAO1 containing reporter plasmid pProbe-dgcH, Cb^*r*^	Wei et al. (2019b)
***Escherichia coli***		
DH5α	*supE44*Δ*lacU169* (Φ80 *lacZ*ΔM15) *recA hsdR17 recA1 endA1 gyrA96 thi-1 relA1*	Lab collection
S17-1	*thi pro hsdR recA;* chromosomal RP4, Tra^+^	Lab collection
***Plasmids***		
pProbe-AT′	Promoterless *gfp* reporter plasmid, Ap^*r*^	Miller et al. (2000)
pProbe-lasR	Promoter sequence of *lasR* inserted into *gfp* reporter plasmid pProbe-AT′, Ap^*r*^	This study
pProbe-mvfR	Promoter sequence of *mvfR* inserted into *gfp* reporter plasmid pProbe-AT′, Ap^*r*^	This study
pProbe-rhlR	Promoter sequence of *rhlR* inserted into *gfp* reporter plasmid pProbe-AT′, Ap^*r*^	This study
pProbe-gacS	Promoter sequence of *gacS* inserted into *gfp* reporter plasmid pProbe-AT′, Ap^*r*^	This study
pProbe-gacA	Promoter sequence of *gacA* inserted into *gfp* reporter plasmid pProbe-AT′, Ap^*r*^	This study
pProbe-cyaB	Promoter sequence of *cyaB* inserted into *gfp* reporter plasmid pProbe-AT′, Ap^*r*^	This study
pProbe-rpoS	Promoter sequence of *rpoS* inserted into *gfp* reporter plasmid pProbe-AT′, Ap^*r*^	This study
p*cupE1*-PUCPgfps	Transcriptional reporter plasmid of *cupE1*, Gm^*r*^	Wang et al. (2019)
p*pprB*-PUCPgfps	Transcriptional reporter plasmid of *pprB*, Gm^*r*^	Wang et al. (2019)
***Primers***		
gacS-proFw	GGAATTCGCAGCATGATGTCCATCAGG	
gacS-proRv	CCCAAGCTTCTTGATGCCGAGATCCTTG	
gacA-proFw	GGAATTCGGAAGCAATCCTGGATCGTCG	
gacA-proRv	CCCAAGCTTGGTGCGTACCAGATCGTGGTC	
cyaB-proFw	GGAATTCCTGGGTGATGCTCAAGGACG	
cyaB-proRv	CCCAAGCTTGGTAGGCTTCATGCGCTGGA	
rpoS-proFw	GGAATTCGCGAGCGGTACTCTGATCG	
rpoS-proRv	CCCAAGCTTCTCCAGGAGGAGCACTTC	
mvfR-proFw	GGAATTCCTACACCTGAAGGCGCAAC	
mvfR-proRv	GGGGTACCGATGACCTGGAGGAACATG	
rhlR-proFw	GGGGTACCCACTGGGAGCCTTGCTGC	
rhlR-proRv	CCCAAGCTTCATCTCGCTACGCAAACC	
lasR-proFw	GGAATTCGTGTGACTGGGTATTCAGTTCG	
lasR-proRv	CCCAAGCTTCGTCAACCAAGGCCATAGC	

### Growth Curve

Overnight cultures of bacterial strains in LB were diluted (1:100) in 3 ml LB medium (30 μl culture into 3 ml LB broth) and precultures incubated aerobically at 37°C in a shaker at 200 rpm to an OD600nm of 0.5. The precultures were further diluted (1:100) in 1 ml LB medium (10 μl precultures in 990 μl LB broth). Growth was then analyzed in 10 × 10-wells microtiter plates containing 294 μl LB medium to which 6 μl of diluted precultures containing 10^5^ cells was added to obtain a final 1:5000 dilution. Control wells contained only the medium without preculture. TRQ treatment was prepared in a 1:4, 1:8, 1:16 dilution using LB medium. The microtiter plates were incubated for 72 h at 37°C in a Bioscreen incubator (Life Technologies, Finland) using the following settings: shaking for 20 s every 3 min and absorbance measured every 30 min at 600 nm. Each culture was prepared in triplicate.

### Virulence Factors Detection

Pyocyanin production was visualized by plating the bacteria on *Pseudomonas*-agar (P-agar or King’s A medium), followed by 48 h incubation. Pyocyanin production resulted in a deep blue coloration of the medium. Quantification of pyocyanin was modified from previous studies (Vinckx et al., 2010). Briefly, the agar was collected in a falcon tube and 10 ml of chloroform was added per 12.5 g of agar medium. The phenazine pigment was extracted during 2 h incubation at 37°C after which 2 ml 0.5 M HCl was added and shaken vigorously. The pink top layer was recovered, and its absorbance was measured at 520 nm.

Other QS-regulated virulence factors including rhamnolipid, elastase, and alkaline protease were detected using methods described previously (Aybey and Demirkan, 2016). TRQ was diluted with an 1/4 ratio in all assay medium and the inhibitory effect was analyzed against untreated controls. All experiments were repeated three times with three technical replicates.

### Transcriptional Expression Analysis

The GFP reporter plasmid pProbe-AT′ was used to construct transcriptional fusions with the corresponding tested promoters (Miller et al., 2000). The promoter region was amplified by PCR using I-5 Hi-Fi DNA Hotstart polymerase (MCLAB). The DNA fragment was digested and cloned into digested pProbe-AT′. The resulting plasmids were mobilized into *P. aeruginosa* by conjugation using *E. coli* S17-1 as the donor strain. Bacteria with reporter fusion were growth at 37°C and data were recorded at 2 h intervals after treatment with TRQ. The promoter activity was evaluated by measuring GFP intensity values captured with Ex/Em 488/520 via calibrating with the corresponding OD_600_ (Synergy H4 Hybrid Reader, BioTek). The gene expression level was expressed as relative GFP intensity.

### Virulence Tests in an Animal Model

Animal virulence test was done according to the previously described method (Moy et al., 2006; Fito-Boncompte et al., 2011). Briefly, *C. elegans* wild-type Bristol strain N2 worms were grown at 20°C on nematode growth medium (NGM) agar plates using *E. coli* OP50 as the nutrient. Synchronous nematode cultures were achieved by exposure to a sodium hypochlorite-sodium hydroxide solution. The resulting eggs were then incubated at 20°C for 24 h to obtain L1 stage worms and transferred to fresh NGM plates to grow until the worms reached L4 life stage. *P. aeruginosa* cells were grown as described above in LB broth overnight and harvested. 10^9^ bacteria cells were spread onto NGM plates and incubated at 37°C overnight. *P. aeruginosa* plates were cooled down to room temperature and 30 L4-synchronized worms infected with *P. aeruginosa* were placed on NGM plates with or without TRQ and incubated at 20°C for bacterial survival assays. *C. elegans* survival data were recorded at a regular interval of each day using an Motic K-400 LED Stereo microscope. Worms were considered dead if they were unresponsive to gentle touch with a platinum wire pick. An infection assay was performed with three independent replicates and repeated three times.

### RNA Extraction

Overnight cultures of *P. aeruginosa* in LB were used to inoculate fresh LB medium in an 1:1000 dilution in the absence and presence of TRQ (1:4 dilution ratio). After 6 h (EXP) and 12 h (STA) of incubation, one ml of culture was immediately fixed with 2 ml of RNA Protect Reagent (Qiagen), following the manufacturer’s instructions, and the fixed cell pellets were frozen at −80°C until further use. All experiments were performed with three technical replicates. Total RNA was extracted using TRIzol^®^ Reagent according to the manufacturer’s instructions (Invitrogen) and genomic DNA was removed using RNase-free DNase I (TaKaRa). Then RNA quality was determined using 2100 Bioanalyzer (Agilent) and quantified using the ND-2000 (NanoDrop Technologies). High-quality RNA sample (OD260/280 = 1.8∼2.2, OD260/230 ≥ 2.0, RIN ≥ 6.5, 28S:18S ≥ 1.0, >10 μg) was used to construct the sequencing library.

### Library Preparation and Illumina Hiseq Sequencing

RNA-seq strand-specific libraries were prepared following TruSeq RNA sample preparation Kit from Illumina (San Diego, CA, United States), using 5 μg of total RNA. Briefly, rRNA was removed by RiboZero rRNA removal kit (Epicenter), fragmented using fragmentation buffer. cDNA synthesis, end repair, A-base addition and ligation of the Illumina-indexed adaptors were performed according to Illumina’s protocol. Libraries were then size selected for cDNA target fragments of 200∼300 bp on 2% Low Range Ultra Agarose followed by PCR amplified using Phusion DNA polymerase (NEB) for 15 PCR cycles. After quantification by TBS380 Mini-Fluorometer, paired-end libraries were sequenced by Shanghai Biozeron Biotechnology Co., Ltd. (Shanghai, China) with the Illumina HiSeq PE 2 × 151 bp read length.

### Reads Quality Control and Mapping

The raw paired end reads were trimmed and quality controlled by Trimmomatic with default parameters (Bolger et al., 2014). Then clean reads were separately aligned to the reference genome (*Pseudomonas aeruginosa* PAO1, Accession number NC_002516) with orientation mode using Rockhopper software (McClure et al., 2013; Tjaden, 2015), which was a comprehensive and user-friendly system for computational analysis of bacterial RNA-seq data. As input, Rockhopper takes RNA sequencing reads generated by high-throughput sequencing technology to calculate gene expression levels with default parameters.

### Differential Expression Analysis and Functional Enrichment

To identify DEGs (differential expression genes) between two different samples, the expression level for each transcript was calculated using the fragments per kilobase of read per million mapped reads (RPKM) method. The method edgeR was used for differential expression analysis (Robinson et al., 2010). The DEGs between two samples were selected using the following criteria: the logarithmic of fold change was greater than 2 and the false discovery rate (FDR) should be less than 0.05. To understand the functions of these differential expressed genes, GO functional enrichment and KEGG pathway analysis were carried out by Goatools (Klopfenstein et al., 2018) and KOBAS (Xie et al., 2011), respectively. DEGs were significantly enriched in GO terms and metabolic pathways when their Bonferroni-corrected *P*-value was less than 0.05. The RNA-seq datasets have been deposited in National Center for Biotechnology Information (NCBI) with an accession number GSE141753.

### Statistical Analysis

The data of virulence factor production, transcriptional analysis, and virulence test were analyzed by one-way ANOVA. Student’s *t*-test was used when one-way ANOVA revealed significant differences (*P* < 0.05). Survival data were analyzed via the Kaplan–Meier method and the log-rank test was used to compare the significant differences between subgroups (*P* < 0.01). All statistical analyses were performed with GraphPad Prism statistical software (GraphPad Software, La Jolla, CA, United States) with the assistance of Excel (Microsoft).

## Results

### TRQ Inhibits the Production of Virulence Factors in *Pseudomonas aeruginosa*

**FIGURE 1:**
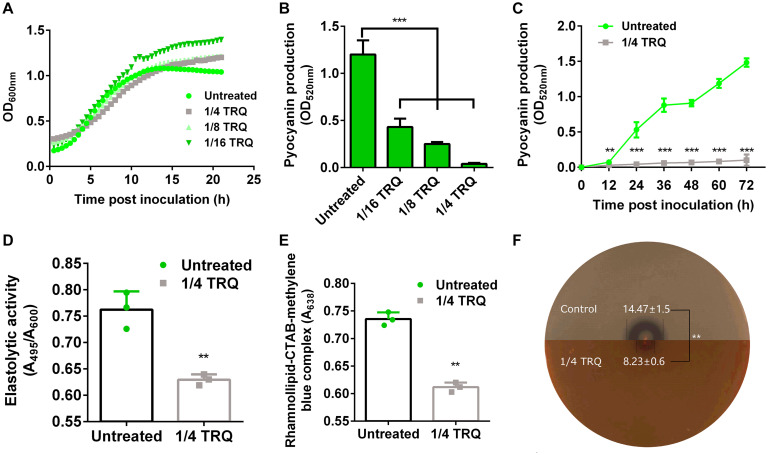
TRQ inhibits the production of virulence factors in *P. aeruginosa.* **(A)** TRQ treatment does not affect *P. aeruginosa* growth. **(B)** TRQ inhibits pyocyanin production in a dose-dependent manner. **(C)** TRQ inhibits pyocyanin production in a time-dependent manner. **(D)** Effect of TRQ on elastase activity. PAO1 was cultivated in LB medium at 37°C for 24 h in the presence of TRQ and then added to elastin-Congo red. Elastolytic activity was performed in elastin-Congo red buffer. **(E)** Effect of TRQ on rhamnolipid production. PAO1 was cultivated in fresh LB broth with and without TRQ at 37°C for 24 h and added to agar-free CTAB medium and incubated for 48 h. The amount of rhamnolipid-CTAB-methylene blue complex biomass in the presence of TRQ was determined. **(F)** Effect of TRQ on alkaline protease activity. The production of alkaline protease was measured using skimmed milk agar plate. A colony from overnight cultures of PAO1 was inoculated using a toothpick at the center of the plates and then incubated at 37°C for 12 h. The production of alkaline protease was confirmed by the formation of a clear zone around bacterial colony. The halo diameters were determined and shown in corresponding region (mm in unit). The upper half plate is untreated control and the bottom half plate is TRQ-containing plate. The dashed lines indicate the halo. Statistical analysis was based on pairwise comparisons (Student’s *t*-test). **P < 0.01; ***P < 0.001. Error bars represent the SD of three replicates.

It has been established that TRQ could affect the viability of bacterial cells (Yang et al., 2018). Therefore, we measured the growth of *P. aeruginosa* PAO1 in the presence or absence of different concentrations of TRQ (1/4, 1/8, 1/16 dilution ratio). As can be seen from Figure 1A, we found that *P. aeruginosa* growth kinetics were unaffected at all tested concentrations, indicating that TRQ formula has no inhibitory effect on planktonic *P. aeruginosa* growth. It was also interesting to notice that when different concentrations of TRQ were added to *P. aeruginosa* cultures, we observed that the production of phenazine pyocyanin was greatly reduced. We further used *Pseudomonas* isolation agar as growth medium to determine the production of pyocyanin and detected that pyocyanin production was significantly decreased in both dose- and time-dependent manner by TRQ treatment (Figures 1B,C). Furthermore, we determined that virulence factors including rhamnolipid (Figure 1D), elastase (Figure 1E) and alkaline protease (Figure 1F) were all significantly repressed by TRQ treatment, indicating TRQ might function as an anti-virulence agent against *P. aeruginosa.*

### TRQ Inhibits the Expression of QS Regulator Genes

**FIGURE 2:**
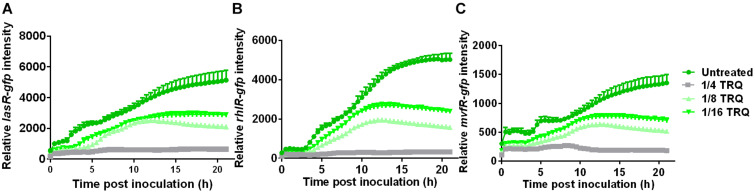
TRQ treatment inhibits the expression of QS regulator genes in *P. aeruginosa*. *las-gfp* expression analysis **(A)**, *rhlR-gfp* expression analysis **(B)**, and *mvfR-gfp* analysis **(C)** by TRQ treatment at different concentrations (untreated control, 1/4 TRQ, 1/8 TRQ, and 1/16 TRQ).

Since the production of virulence factors such as pyocyanin are under the control of QS systems in *P. aeruginosa*, we therefore determined whether the expression of these QS systems was affected by TRQ treatment. Three QS signal receptors were chosen and their transcriptional fusions with GFP reporters were constructed to probe the relative expression level of these QS systems. As can be seen from Figure 2, the transcriptional expression of *lasR*, *rhlR*, and *mvfR* was all greatly repressed by TRQ treatment in a dose-dependent manner. Our results confirmed that TRQ could indeed inhibit the expression of QS systems in *P. aeruginosa* and thus inhibit the production of virulence factors such as pyocyanin.

### Transcriptome Analyses Unravel the Mode of Action of TRQ

**FIGURE 3:**
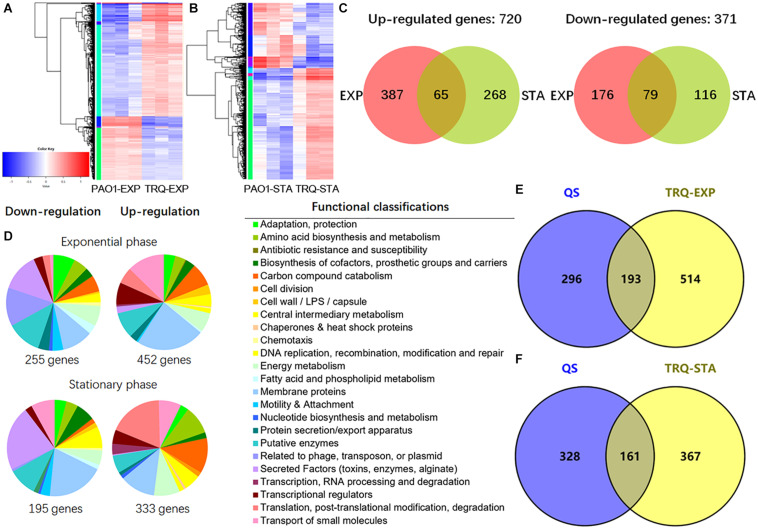
Transcriptome analyses of TRQ-treated *P. aeruginosa* cells. **(A)** Hierarchical clustering and heatmap analysis of the differentially expressed genes of TRQ-treated *P. aeruginosa* cells under exponential growth phase analysis (PAO1-EXP vs. TRQ-EXP) and stationary growth phase (PAO1-STA vs. TRQ-STA) **(B)**. **(C)** Comparison of up-regulated and down-regulated genes in TRQ-treated *P. aeruginosa* cells in the exponential growth phase (EXP) and in stationary growth phase (STA). **(D)** Pie charts showing the relative abundance of functional classifications of differentially expressed genes of TRQ-treated *P. aeruginosa* under two growth conditions. Hypothetical genes were removed from the pies to ensure ease of reading. **(E)** TRQ-treated *P. aeruginosa* regulon shared overlap with custom QS regulon. Venn diagrams showing the overlap between QS regulon and TRQ-treated *P. aeruginosa* cells in the exponential growth phase and in the stationary growth phase **(F)**.

To gain insight into the regulatory breadth of TRQ treatment on *P. aeruginosa*, we carried out a global RNA-seq analysis of the transcriptional responses to determine the number of genes under both exponential (EXP) and stationary (STA) growth phases. *P. aeruginosa* cells were harvested, and RNA was extracted and processed according to the recommendations of the Illumina system for RNA-seq analyses. After data qualification control and processing, we finally obtained a comprehensive data set summarized in Supplementary Tables S1, S2. Clearly, PAO1 and TRQ-treated PAO1 demonstrated distinct gene expression profiles according to the heat map diagram (Figures 3A,B). A large number of genes were differentially expressed under both EXP and STA growth phases and showed overlap between these two conditions. In total, 371 genes were downregulated and 720 genes were upregulated in EXP and STA growth phases by TRQ treatment (Figure 3C). Functional classification analysis showed that genes encoding secreted factors and membrane proteins were largely represented as downregulated under both EXP and STA growth phases by TRQ treatment. In contrast, genes encoding carbon compound metabolism, transport of small molecules, and translation and post-translation modification were mostly up-regulated under EXP and STA growth phases (Figure 3D).

As can be seen from Table 2, the expression of a large number of virulence genes was downregulated upon TRQ treatment, including these QS-regulated genes. Specifically, PQS signal synthase operon (*pqsBCDE*), hydrogen cyanide synthase operon (*hcnABC*), two phenazine biosynthesis operons (*phzA1B1C1D1F1G1* and *phzA2B2C2D2F2G2*), *lasA*, *lasB*, *lecB*, *lecA*, *rhlAB*, *chiC*, alkaline protease operon (*aprXDEFAI*) were highly repressed upon TRQ treatment compared to non-treatment control. This finding was confirmed by phenotypic analysis (Figure 1B) and transcriptional analyses of QS regulator genes (Figure 2B) and further showed that all three QS systems in *P. aeruginosa* were inhibited by TRQ treatment. In addition, genes involved in type VI secretion (T6SS) were also downregulated by TRQ treatment.

**TABLE 2:** Selected downregulated genes upon TRQ treatment.

		Sampling time^*c*^			
ORF^*a*^	Gene^*b*^	EXP	STA	QS^*d*^	Protein description^*e*^
PA0122	*rahU*	−3.6	−2.7	+	RahU
PA0284		1.8	−1.7		Hypothetical protein
PA0713		−1.5	−1.9	+	Hypothetical protein
PA0852	*cbpD*	−1.6	−3.8	+	Chitin-binding protein CbpD precursor
PA0852.1		−1.6	−3.8		Uncharacterized protein
PA0977			−1.5		Hypothetical protein
PA0997	*pqsB*	−4.1	−1.8	+	PqsB
PA0998	*pqsC*	−4.2	−1.7	+	PqsC
PA0999	*pqsD*	−4.1	−1.6	+	3-Oxoacyl-[acyl-carrier-protein] synthase III
PA1000	*pqsE*	−3.8	−1.5	+	Quinolone signal response protein
PA1216		−2.6	−2.2	+	Hypothetical protein
PA1217		−2.5	−1.8	+	Probable 2-isopropylmalate synthase
PA1218		−2.3	−1.4	+	Hypothetical protein
PA1245	*aprX*		−1.8	+	AprX
PA1246	*aprD*		−1.4	+	Alkaline protease secretion protein AprD
PA1247	*aprE*		−1.6	+	Alkaline protease secretion protein AprE
PA1248	*aprF*		−1.4	+	Alkaline protease secretion outer membrane protein AprF precursor
PA1249	*aprA*		−3.1	+	Alkaline metalloproteinase precursor
PA1250	*aprI*		−1.4	+	Alkaline proteinase inhibitor AprI
PA1384	*galE*		−1.3		UDP-glucose 4-epimerase
PA1784		−1.2	−1.6	+	Hypothetical protein
PA1837			−2		Hypothetical protein
PA1838	*cysI*		−1.5		Sulfite reductase
PA1869	*acp1*	−2.4	−2.2	+	Acp1
PA1871	*lasA*	−2	−3.4	+	LasA protease precursor
PA1899	*phzA2*	−2.1	−2.8		Probable phenazine biosynthesis protein
PA1900	*phzB2*	−3.2	−5.6		Probable phenazine biosynthesis protein
PA1901	*phzC2*	−2.8	−3.7	+	Phenazine biosynthesis protein PhzC
PA1902	*phzD2*	−4	−4	+	Phenazine biosynthesis protein PhzD
PA1903	*phzE2*	−3.9	−3.5	+	Phenazine biosynthesis protein PhzE
PA1904	*phzF2*	−3.7	−3.5	+	Probable phenazine biosynthesis protein
PA1905	*phzG2*	−3.3	−3.4	+	Probable pyridoxamine 5′-phosphate oxidase
PA1927	*metE*		−1.3	+	5-methyltetrahydropteroyltriglutamate-homocysteine S-methyltransferase
PA2030			−1.6	+	Hypothetical protein
PA2031			−1.7	+	Hypothetical protein
PA2062			−2.4		Probable pyridoxal-phosphate dependent enzyme
PA2066		−1.1	−1.6	+	Hypothetical protein
PA2067		−1.4	−1.8	+	Probable hydrolase
PA2068		−2.6	−1.9	+	Probable major facilitator superfamily (MFS) transporter
PA2069		−2.8	−3.7	+	Probable carbamoyl transferase
PA2193	*hcnA*	−2.3	−3	+	Hydrogen cyanide synthase HcnA
PA2194	*hcnB*	−2.2	−2.9	+	Hydrogen cyanide synthase HcnB
PA2195	*hcnC*	−2.4	−3	+	Hydrogen cyanide synthase HcnC
PA2204		1.6	−2.1		Probable binding protein component of ABC transporter
PA2274			−2.2	+	Hypothetical protein
PA2300	*chiC*	−2.6	−4.5	+	Chitinase
PA2302	*ambE*		−2.1	+	AmbE
PA2303	*ambD*		−2.1	+	AmbD
PA2304	*ambC*		−2.1	+	AmbC
PA2305	*ambB*		−2	+	AmbB
PA2328		−1.5	−1.8	+	Hypothetical protein
PA2329		−1.6	−2.3	+	Probable ATP-binding component of ABC transporter
PA2330		−1.7	−2.7	+	Hypothetical protein
PA2331		−1.5	−3.3	+	Hypothetical protein
PA2381		−1	−2.5		Hypothetical protein
PA2459			−1.4		Hypothetical protein
PA2564		−2.3	−1.5	+	Hypothetical protein
PA2565		−2.3	−1.5	+	Hypothetical protein
PA2566		−2.3	−1.7	+	Conserved hypothetical protein
PA2570	*lecA*	−3.5	−3.6	+	LecA
PA2588			−1.7	+	Probable transcriptional regulator
PA3361	*lecB*	−5.6	−3.3	+	Fucose-binding lectin PA-IIL
PA3450	*lsfA*		−2.1		1-Cys peroxiredoxin LsfA
PA3478	*rhlB*	−3.2	−3.3	+	Rhamnosyltransferase chain B
PA3479	*rhlA*	−3.7	−3.8	+	Rhamnosyltransferase chain A
PA3520		−2.2	−2.4	+	Hypothetical protein
PA3724	*lasB*	−1.7	−1.7	+	Elastase LasB
PA3734		−2.3	−1.6	+	Hypothetical protein
PA3813	*iscU*		−1.4		Probable iron-binding protein IscU
PA3814	*iscS*		−1.3		L-Cysteine desulfurase (pyridoxal phosphate-dependent)
PA3869			−1.4		Hypothetical protein
PA3884			−1.4		Hypothetical protein
PA3928		−1.4	−1.8		Hypothetical protein
PA3929	*cioB*	−1.2	−1.9		Cyanide insensitive terminal oxidase
PA3930	*cioA*	−1.4	−2.1		Cyanide insensitive terminal oxidase
PA4078		−2.1	−3.9	+	Probable non-ribosomal peptide synthetase
PA4079			−1.4		NaD(P)H-dependent carbonyl reductase
PA4129		−2.1	−2.1	+	Hypothetical protein
PA4130		−2.5	−2	+	Probable sulfite or nitrite reductase
PA4131		−2.3	−1.9	+	Probable iron–sulfur protein
PA4132		−1.9	−1.9	+	Conserved hypothetical protein
PA4133		−4.2	−2.5	+	Cytochrome c oxidase subunit (cbb3-type)
PA4134		−4.1	−2.3	+	Hypothetical protein
PA4141		−2.5	−2.5	+	Hypothetical protein
PA4205	*mexG*	1.3	−3.5	+	Hypothetical protein
PA4206	*mexH*		−2	+	Probable resistance-nodulation-cell division efflux membrane protein
PA4208	*opmD*		−1.3	+	Probable outer membrane protein precursor
PA4209	*phzM*	−3.7	−3.2	+	Probable phenazine-specific methyltransferase
PA4210	*phzA1*	−3.6	−2.2	+	Probable phenazine biosynthesis protein
PA4211	*phzB1*	−5.8	−4.9	+	Probable phenazine biosynthesis protein
PA4212	*phzC1*	−2.9	−3.7	+	Phenazine biosynthesis protein PhzC
PA4213	*phzD1*	−4	−4	+	Phenazine biosynthesis protein PhzD
PA4214	*phzE1*	−3.9	−3.5	+	Phenazine biosynthesis protein PhzE
PA4215	*phzF1*	−3.7	−3.5	+	Probable phenazine biosynthesis protein
PA4216	*phzG1*	−3.4	−3.4	+	Probable pyridoxamine 5′-phosphate oxidase
PA4217	*phzS*	−4.3	−3.8	+	Flavin-containing monooxygenase
PA4442	*cysN*	1.7	−1.9	+	ATP sulfurylase GTP-binding subunit/APS kinase
PA5220		−2.1	−1.8	+	Hypothetical protein

*^*a*^PA numbers are from http://www.pseudomonas.com; ORF, open reading frame. ^*b*^Genes are obtained from http://www.pseudomonas.com. ^*c*^The ratios (log2 ratio) represent expression levels in PAO1 cultures supplemented with 1/4 TRQ relative to that of the control at exponential phase (EXP) and stationary phase (STA). ^*d*^These genes were identified as QS regulated in previous transcriptome studies. ^*e*^Proteins are described from http://www.pseudomonas.com.*

Given the inhibition of QS systems, we aimed to find out the overlap between TRQ-treated *P. aeruginosa* regulon and QS regulon under typical growth conditions (LB medium in 500 mL Erlenmeyer flasks, grow at 37°C and shake at 200 rpm). We then integrated the previously identified QS regulon into a custom made QS regulon (Hentzer et al., 2003; Schuster et al., 2003) and compared it with the TRQ-treated *P. aeruginosa* regulon. We found that our determined regulons (TRQ-EXP and TRQ-STA) showed a close relationship with custom QS regulon (Figures 3E,F). More than 193 (TRQ-EXP) and 161 (TRQ-STA) genes were identified to overlap with the custom QS regulon (489 genes) in *P. aeruginosa*, suggesting that TRQ treatment has a considerable impact on the regulation of QS and clearly this dis-regulation of QS systems will contribute to the reduced virulent phenotypes.

Altogether, through our extensive RNA-seq analyses, we can conclude that the mode of action of TRQ is closely linked to the inhibition of QS systems in *P. aeruginosa*.

### Component Analyses of TRQ Reveal Sub-inhibitory Effect on QS Regulator Genes

**FIGURE 4:**
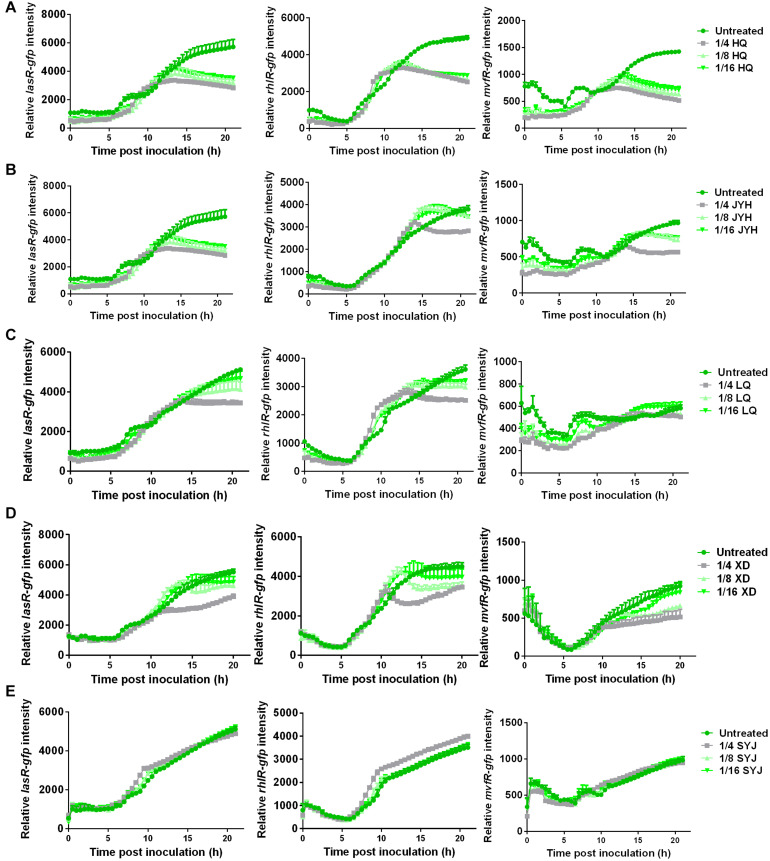
Component analyses of TRQ reveal sub-inhibitory effects on QS regulator genes. The same conditions were used as Figure 2. The left panel showed the expression analysis of *lasR-gfp*; the middle panel showed the expression of *rhlR-gfp*; the right panel showed the expression of *mvfR-gfp*. Five components in TRQ formula were tested individually for the gene expression analyses: **(A)** HQ; **(B)** JYH; **(C)** LQ; **(D)** XD; **(E)** SYJ.

TRQ formula consists of five different components [Huang Qin (HQ), Jin Yin Hua (JYH), Lian Qiao (LQ), Xiong Dan (XD), and Shan Yang Jiao (SYJ)] (Yang et al., 2018). To further understand the roles played by these components, we used individual component to determine their effects on the expression of QS regulatory genes. As can be seen from Figures 4A–D, we showed that HQ, JYH, LQ, and XD had an inhibitory effect on the expression of all three QS regulator genes. To our surprise, SYJ demonstrated only little inhibitory effect on the expression of QS regulator genes (Figure 4E).

**FIGURE 5:**
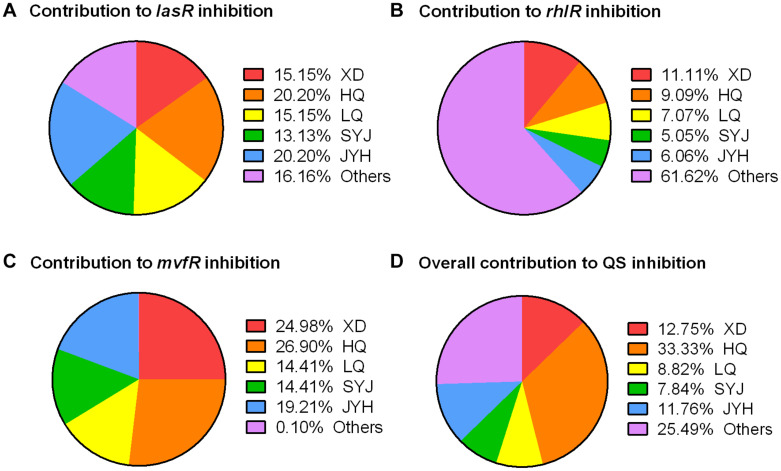
Contribution of five components in TRQ formula to QS inhibition. Contribution to *lasR* inhibition **(A)**, *rhlR* inhibition **(B)**, *mvfR* inhibition **(C)**, and overall inhibition **(D)**. Others indicate the mixing effect of five components in TRQ. Fixed data points were extracted from each treatment and calculated as the percentage calibrated by total inhibition (100%).

To further understand the exact roles of these five components within TRQ formula, we calculated the individual contribution to the inhibition of QS regulatory genes. As shown in Figure 5, the inhibition of *lasR* expression depended on all five components with the contribution of unknown mixing effects (indicated as others in Figure 5A). The inhibition of *rhlR* expression depended mainly on the unknown mixing effects, and the five components showed minor effects on *rhlR* expression (Figure 5B). As for *mvfR* expression, HQ demonstrated the most significant effect and the mixing only showed a marginal effect on *mvfR* expression, indicating that these five components were sufficient for *mvfR* inhibition (Figure 5C).

Altogether, we can conclude that all five components had inhibitory effects on QS systems in *P. aeruginosa* and, in particular HQ showed the most efficient role in QS inhibition with the assistance of the other four ingredients (Figure 5D).

### TRQ Inhibits QS Systems via Repression of Two-Component Systems GacS/GacA and PprA/PprB in *P. aeruginosa*

**FIGURE 6:**
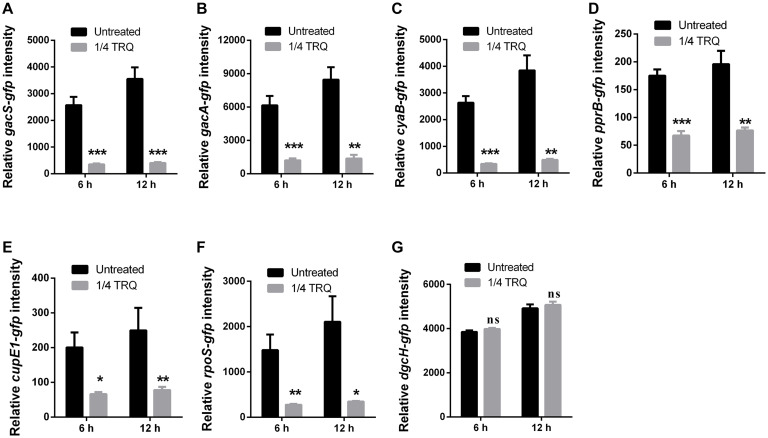
Upstream genetic determinants of QS systems impacted by TRQ treatment. **(A)** *gacS-gfp*; **(B)** *gacA-gfp*; **(C)** *cyaB-gfp*; **(D)** *pprB-gfp*; **(E)** *cupE1-gfp*; **(F)** *rpoS-gfp*; **(G)** *dgcH-gfp*. Relative fluorescence intensity was defined by GFP intensity calibrated with OD600nm. Statistical analysis was based on pairwise comparisons (Student’s *t*-test). ***P < 0.001; **P < 0.01; *P < 0.05; ns, non-significant. Error bars represent the SD.

Given that TRQ formula significantly attenuates the expression of QS systems in *P. aeruginosa*, we hypothesized whether there were upstream genetic determinants targeted by TRQ treatment. Previously, it was reported that two-component system (TCS) GacS/GacA positively regulated the expression of *lasR* expression in *P. aeruginosa* (Venturi, 2006). In addition, Vfr was also demonstrated to positively regulate the expression of global virulence factors and Vfr-dependent transcription was linked with its cofactor, cyclic AMP (cAMP), which is synthesized by two adenylate cyclases, CyaA (PA5272) and CyaB (PA3217) (Almblad et al., 2015). Based on these information, we generated transcriptional fusion reporter strains for *gacS*, *gacA* and *cyaB* and detected their transcriptional responses after TRQ treatment. As shown in Figures 6A–C, we could clearly observe that TRQ could reduce the expression of the TCS GacS/GacA and the *cyaB* adenylate cyclase gene, implying that TRQ could regulate these upstream genetic determinants of QS systems.

Additionally, the two-component response regulator PprB was demonstrated to modulate the QS-regulated genes in *P. aeruginosa*, especially the PQS-mediated QS system (de Bentzmann et al., 2012). Furthermore, the TCS PprAB could trigger the hyper-biofilm phenotype with a unique adhesive signature made of BapA adhesin and CupE fimbriae (Giraud et al., 2011; de Bentzmann et al., 2012). More recently, the environmental signal for the TCS PprAB was found to be carbon starvation stress in *P. aeruginosa* and this response was dependent on the sigma factor RpoS (Wang et al., 2019). Therefore, we checked whether the TCS PprAB could be involved in TRQ-mediated QS inhibition. As can be seen from Table 3, RNA-seq analysis indicated that a large array of genes involved in carbon source metabolism were upregulated by TRQ treatment (Table 3), while the transcriptional expression of the TCS sensor kinase gene *pprA* was down-regulated by TRQ treatment (−1.6-fold reduction, log2 ratio, Supplementary Table S1). In addition, TRQ treatment was found to downregulate the expression of several PprAB-regulated genes including the *cupE* operon and *tad* operons (Supplementary Table S1). Therefore, it would be interesting to determine whether excess supply of carbon sources from the TRQ formula would repress the expression of *pprAB.* To this end, we used the transcriptional fusion of *pprB*, *cupE1* and *rpoS* to verify our hypothesis. By adding TRQ to cultures of PAO1 derivatives, we could observe that the expression of these genes was significantly downregulated (Figures 6D–F). As a negative control, we used the previously characterized *dgcH* promoter fusion, which showed invariable under various environmental conditions and genetic backgrounds (Wei et al., 2019b). No significant change was observed in gene expression level (Figure 6G), consistent with its original RNA-seq data (see GEO dataset GSE141753).

**TABLE 3:** Selected upregulated genes upon TRQ treatment.

		Sampling time^*c*^			
ORF^*a*^	Gene^*b*^	EXP	STA	QS^*d*^	Protein description^*e*^
**Amino acid biosynthesis and metabolism**					
PA0025	*aroE*	1.3			Shikimate dehydrogenase
PA0245	*aroQ2*	3.6			3-Dehydroquinate dehydratase
PA0331	*ilvA1*	1.2			Threonine dehydratase, biosynthetic
PA0390	*metX*	1.7			Homoserine *O*-acetyltransferase
PA0782	*putA*	1.8			Proline dehydrogenase PutA
PA0895	*aruC*	1.4			N2-succinylornithine 5-aminotransferase (SOAT)
PA0896	*aruF*	1.2			Subunit I of arginine N2-succinyltransferase
PA0897	*aruG*	1.2			Subunit II of arginine N2-succinyltransferase
PA1818	*cadA*	1.2			Lysine decarboxylase
PA3082	*gbt*	1.2			Glycine betaine transmethylase
PA4731	*panD*	1.4	1.2		Aspartate 1-decarboxylase precursor
**Carbon compound catabolism**					
PA0153	*pcaH*	3.9			Protocatechuate 3,4-dioxygenase, beta subunit
PA0154	*pcaG*	4.1			Protocatechuate 3,4-dioxygenase, alpha subunit
PA0226		3.5	1.6		Probable CoA transferase, subunit A
PA0227		3.3			Probable CoA transferase, subunit B
PA0232	*pcaC*	1.4	1.3		Gamma-carboxymuconolactone decarboxylase
PA0247	*pobA*	1.7			*P*-Hydroxybenzoate hydroxylase
PA2261		2.5		+	Probable 2-ketogluconate kinase
PA2323	*gapN*	2.7			GapN
PA2507	*catA*	2.1	3.4		Catechol 1,2-dioxygenase
PA2508	*catC*	2.0	3.3		Muconolactone delta-isomerase
PA2509	*catB*	1.5	1.4		Muconate cycloisomerase I
PA2515	*xylL*	3.2			*Cis*-1,2- dihydroxycyclohexa3-,4-diene carboxylate dehydrogenase
PA2517	*xylY*	4.1	2.8		Toluate 1,2-dioxygenase beta subunit
PA2518	*xylX*	4.5	2.7		Toluate 1,2-dioxygenase alpha subunit
PA4091	*hpaA*	4.7			4-Hydroxyphenylacetate 3-monooxygenase large chain
PA4092	*hpaC*	4.5			4-Hydroxyphenylacetate 3-monooxygenase small chain
PA4123	*hpcC*	4.2			5-Carboxy-2-hydroxymuconate semialdehyde dehydrogenase
PA4124	*hpcB*	4.0			Homoprotocatechuate 2,3-dioxygenase
PA4125	*hpcD*	4.0			5-Carboxymethyl-2-hydroxymuconate isomerase
PA4127	*hpcG*	3.8			2-Oxo-hept3–ene-1,7-dioate hydratase
PA5351	*rubA1*	1.4			Rubredoxin 1
PA4670	*prs*	1.6	2.0		Ribose-phosphate pyrophosphokinase
PA2862	*lipA*	1.8	2.7		Lactonizing lipase precursor
PA3363	*amiR*	1.5	1.0		Aliphatic amidase regulator
**Central intermediary metabolism**					
PA0654	*speD*	1.5			*S*-Adenosylmethionine decarboxylase proenzyme
PA2393		1.6			Putative dipeptidase
PA3182	*pgl*	1.8		+	6-Phosphogluconolactonase
PA3582	*glpK*	1.5	2.2		Glycerol kinase
PA4100		1.3			Probable dehydrogenase
PA4956	*rhdA*	1.3			Thiosulfate:cyanide sulfurtransferase
PA5435		2.3	1.3		Probable transcarboxylase subunit
PA5436		2.7	1.1		Probable biotin carboxylase subunit of a transcarboxylase
PA4442	*cysN*	1.7	−1.9	+	ATP sulfurylase GTP-binding subunit/APS kinase
PA4443	*cysD*	2.1	−1.1	+	ATP sulfurylase small subunit
PA3181		1.6		+	2-Keto3–deoxy-6-phosphogluconate aldolase
PA4748	*tpiA*	1.2			Triosephosphate isomerase
PA3562	*fruI*	1.3			Phosphotransferase system transporter enzyme I, FruI
**Energy metabolism**					
PA0519	*nirS*	1.7	1.9		Nitrite reductase precursor
PA0521		2.0			Probable cytochrome c oxidase subunit
PA0523	*norC*	4.8			Nitric-oxide reductase subunit C
PA0524	*norB*	4.3			Nitric-oxide reductase subunit B
PA0525		3.1			Probable denitrification protein NorD
PA1317	*cyoA*	1.5	−1.3	+	Cytochrome *o* ubiquinol oxidase subunit II
PA2382	*lldA*	2.1			L-Lactate dehydrogenase
PA2664	*fhp*	3.0			Flavohemoprotein
PA3392	*nosZ*	2.9		+	Nitrous-oxide reductase precursor
PA3393	*nosD*	1.9		+	NosD protein
PA3872	*narI*	2.0		+	Respiratory nitrate reductase gamma chain
PA3873	*narJ*	1.8		+	Respiratory nitrate reductase delta chain
PA3874	*narH*	1.8		+	Respiratory nitrate reductase beta chain
PA3875	*narG*	2.2		+	Respiratory nitrate reductase alpha chain
PA2321	*gntK*	3.1		+	GntK
PA2516	*xylZ*	4.1	1.7		Toluate 1,2-dioxygenase electron transfer component
PA3183	*zwf*	2.1		+	Glucose-6-phosphate 1-dehydrogenase
PA3194	*edd*	1.5		+	Phosphogluconate dehydratase
PA3195	*gapA*	2.5		+	Glyceraldehyde 3-phosphate dehydrogenase
PA3394	*nosF*	1.6		+	NosF protein

*^*a*^PA numbers are from http://www.pseudomonas.com; ORF, open reading frame. ^*b*^Genes are obtained from http://www.pseudomonas.com. ^*c*^The ratios (log2 ratio) represent expression levels in PAO1 cultures supplemented with 1/4 TRQ relative to that of the control at exponential phase (EXP) and stationary phase (STA). ^*d*^These genes were identified as QS regulated in previous transcriptome studies. ^*e*^Proteins are described from http://www.pseudomonas.com.*

Altogether, we could draw the conclusion that TRQ formula functions through at least two TCSs to downregulate QS systems in *P. aeruginosa*.

### TRQ Attenuates the Virulence of *P. aeruginosa* in an Animal Model

**FIGURE 7:**
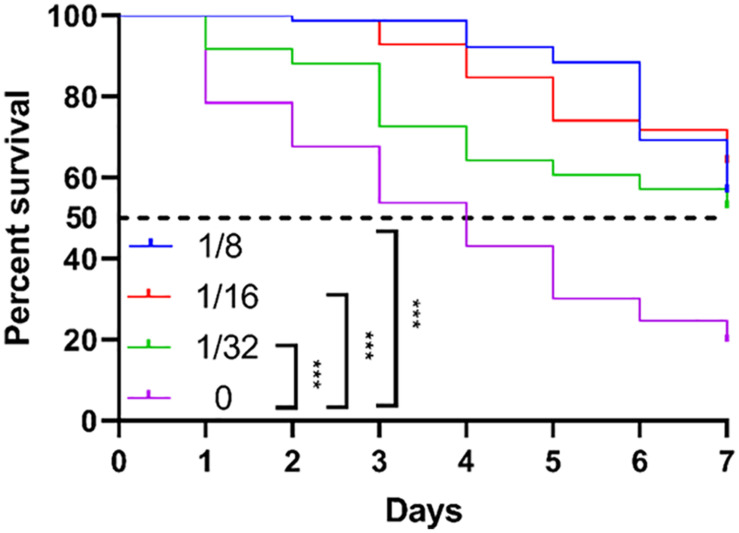
TRQ attenuates the virulence of *P. aeruginosa* toward *C. elegans*. The survival curve was determined by counting the percent of live worms within 7 days. The log-rank test was used to compare the significant differences between subgroups. ***P < 0.001.

As we already observed that TRQ mainly targeted the QS regulatory systems in *P. aeruginosa* which mediates the pathogenesis in plant and animal models via the production of several virulence factors (Mahajan-Miklos et al., 1999; Rahme et al., 2000). We therefore checked the *in vivo* protection role of TRQ against *P. aeruginosa* infection. We fed *C. elegans* with TRQ-treated *P. aeruginosa* cells and then checked the survival rate of worms. As shown in Figure 7, we found that *P. aeruginosa* wild type strain was toxic to the worms since more than 50% of the population died after 4 days and 80% after 7 days. In contrast, TRQ-treated *P. aeruginosa* cells caused a significant increase in worm survival and this effect was TRQ concentration dependent. After 7 days, there were still more than 50% worms alive. Altogether, we demonstrated that TRQ could protect *C. elegans* from killing by *P. aeruginosa*.

## Discussion

*Pseudomonas aeruginosa* is one of the notorious human opportunistic pathogens that cause significant mortality and morbidity in immunocompromised patients. The pathogenicity of *P. aeruginosa* is largely dependent on the production of virulence factors such as pyocyanin, siderophores, elastases, and proteases, which are elaborately controlled by a hierarchal QS regulatory network (Papenfort and Bassler, 2016). This elegant regulatory strategy has attracted extensive attention to develop therapeutics against *P. aeruginosa*-related infections, particularly acute infections. A wide array of natural and synthetic compounds targeting QS systems have been developed and tested *in vitro* and *in vivo* (Starkey et al., 2014; Asfour, 2018; Dogan et al., 2019). For example, furanone C-30 (Hentzer et al., 2002), gingerol (Kim et al., 2015) and garlic extract (Rasmussen et al., 2005) have been reported to possess anti-QS activities. Recently, it has been demonstrated that ginkgolic acids from *Pistacia lentiscus* fruits inhibit *P. aeruginosa* virulence by targeting the *pqs* system (Tahrioui et al., 2020). Particularly, many traditional Chinese medicines have been shown to exert anti-QS and anti-virulence activities. However, the exact mechanism of QS inhibition seems to be still uncertain.

Previously, we have shown that TRQ could be used in synergy with traditional antibiotics to enhance its antibacterial effects against methicillin-resistant *Staphylococcus aureus* (Yang et al., 2018). Furthermore, we have demonstrated that TRQ formula could efficiently eradicate *S. aureus* biofilms in synergy with penicillin (Wang et al., 2011). However, the underlying mechanisms concerning the antibacterial and anti-biofilm activities of TRQ are still poorly understood. In this study, we focused on the Gram-negative bacterium *P. aeruginosa* as a model to investigate the mode of action of TRQ against bacterial infections. Surprisingly, we found that TRQ could target all three QS systems in *P. aeruginosa*, including *las*, *rhl*, and *pqs* (Lee et al., 2013). Through extensive bioinformatic analyses, we found that there was a significant overlap between differentially expressed genes in the exponential and stationary growth phase, suggesting that TRQ has prolonged effects on *P. aeruginosa* transcriptional response. Most of the QS-regulated genes have been found to be repressed by TRQ treatment, including two phenazine biosynthesis operons, *lasA*, *lasB*, *lecA*, *lecB*, *rhlAB*, *chiC*, and the alkaline protease operon (*aprXDEFAI*).

Interestingly, we also observed that the expression of *pqsE* was repressed in both exponential and stationary growth phases (Table 2). Recently, PqsE was reported to function as an alternative ligand synthase pairing with the RhlR receptor to control virulence and biofilm formation in *P. aeruginosa* (Mukherjee et al., 2018). The reduced *pqsE* gene expression would therefore disrupt the RhlR-mediated virulence gene expression. Additionally, the down-regulation of *pqsBCDE* will lead to quenching of the *pqs* system in *P. aeruginosa* and further contribute to reduced virulence (Deziel et al., 2005; Tahrioui et al., 2020). Collectively, all these evidences indicated that TRQ treatment could effectively target the *pqs* system and subsequently contribute to the inhibition of QS systems in *P. aeruginosa*.

The inhibition of *rhl* by TRQ might involve a complex mechanism since much less contribution (less than 40%) of the five components in TRQ has been observed, while unknown mixing effects play significant roles in *rhlR* suppression. In addition, the lack of RhlR structural information has proved the difficulty to analyze the protein-ligand interaction and to develop structure-function based therapeutics for this regulatory protein. However, several progresses have been made recently. For example, it was reported that meta-bromo-thiolactone (mBTL) could target both LasR and RhlR *in vitro*, while RhlR is the relevant *in vivo* target (O’Loughlin et al., 2013). Furthermore, RhlR responds to a suggested PqsE-encoded alternative ligand in addition to its canonical C4-HSL to promote a transcriptional program in the absence of RhlI (Mukherjee et al., 2017, 2018). Most recently, mBTL-complexed RhlR has allowed the first purification of RhlR protein (McCready et al., 2019). All these new findings will facilitate the understanding of *rhl* QS system and the future study of its inhibition mechanism.

The component contributions to QS inhibition analyses have shed light on the underlying mechanism of each component of TRQ and showed that all five components are necessary for the efficient and complete inhibition of QS systems, since we have noticed that none of each separate component could completely inhibit the expression of QS regulator genes. In addition, the unknown mixing effects of these five components are still not well understood. Therefore, in the future, we should focus on deciphering the individual contribution of TRQ components to determine the exact chemical mechanism within these five components, although we have already obtained the results that at least the HQ played the most important role in the inhibition of QS systems in our test conditions. It is interesting to note that multiple ingredients have been characterized within HQ and their potential involvement in TCS or QS inhibition has been reported (Supplementary Table S4). For instance, one of the HQ ingredients, scutellarein, might be involved in the inhibition of protein kinase activity (You et al., 2017). It is tempting to investigate the underlying mechanisms of action in future studies.

Our RNA-seq analyses also enabled us to further understand the upstream genetic determinants that mediate the inhibition of QS systems in *P. aeruginosa*. We are particularly interested in the finding that the TCSs GacS/GacA and PprA/PprB were downregulated by TRQ treatment, possibly through carbon source supply due to the existence of two nutrients within TRQ (i.e., SYJ and XD), which contain excess amounts of amino acids and other nutritional ingredients. The TCS PprA/PprB was previously shown to control some QS-regulated genes, CupE fimbriae expression and thus biofilm formation (Giraud et al., 2011), although we have not systematically examined the effect of TRQ on the formation of biofilms in *P. aeruginosa* in this study. Most recently, this TCS was shown to be triggered by carbon starvation stress and this process was dependent on the sigma factor RpoS (Wang et al., 2019). As for the TCS GacS/GacA, it was previously known that certain QS inhibitors could function through this pathway to repress the expression of QS-regulated genes in *P. aeruginosa* (Tan et al., 2014). Altogether, our results shed light on the multilevel inhibitory mechanisms of TRQ on the regulatory cascades in *P. aeruginosa*.

One of the technical issues concerning our study is that we used transcriptional fusion rather than qRT-PCR to validate our RNA-seq data. In principle, the transcriptional fusion analysis takes advantage of the accumulation of GFP fluorescence and serves as a reporter for the rate of transcription initiation from the promoter region. In contrast, RNA-seq, DNA microarray, and qRT-PCR report mRNA concentration, which is a balance between mRNA production and degradation (Zaslaver et al., 2006). We also realized that the transcriptional fusions are indeed plasmid-based constructs, therefore with increased copy number. Since the genes in fusion with GFP represent regulators which are not highly expressed (Supplementary Table S3), the advantage of using plasmid-borne fusions is evident. Specifically, the reason that these genes were not included in Table 2 and Supplementary Tables S1, S2 was due to their low fold change (between −2 and 2). Therefore, it appears that we did not detect them in the RNA-seq with stringent filtering. On the other hand, a factor two increase in transcripts of a regulator can have an effect more pronounced on the downstream genes, which is the case of the upstream targets by TRQ treatment. This is also the advantage of combining different methods and the reason we could identify the upstream genetic regulators that affect QS systems in our study.

In addition to inhibition of regulatory gene expression, TRQ might function through other mechanisms that would involve the possible interference with ligand-receptor interactions. For example, we have recently shown that one of the Chinese medicinal herb extracts (MHE) could interrupt the binding of MvfR to the *pqsA* promoter region, thus inhibiting the virulence factors production. Chemical analysis and molecular docking analysis showed that butyrolactone (BTL) and furfuryl alcohol (FA) within MHE could function as interference molecule(s) with the PQS signal to compete with MvfR (Wei et al., 2019a). Therefore, it would be interesting to identify the chemical components within TRQ to test for their performance and involvement in ligand-receptor binding interference.

**FIGURE 8:**
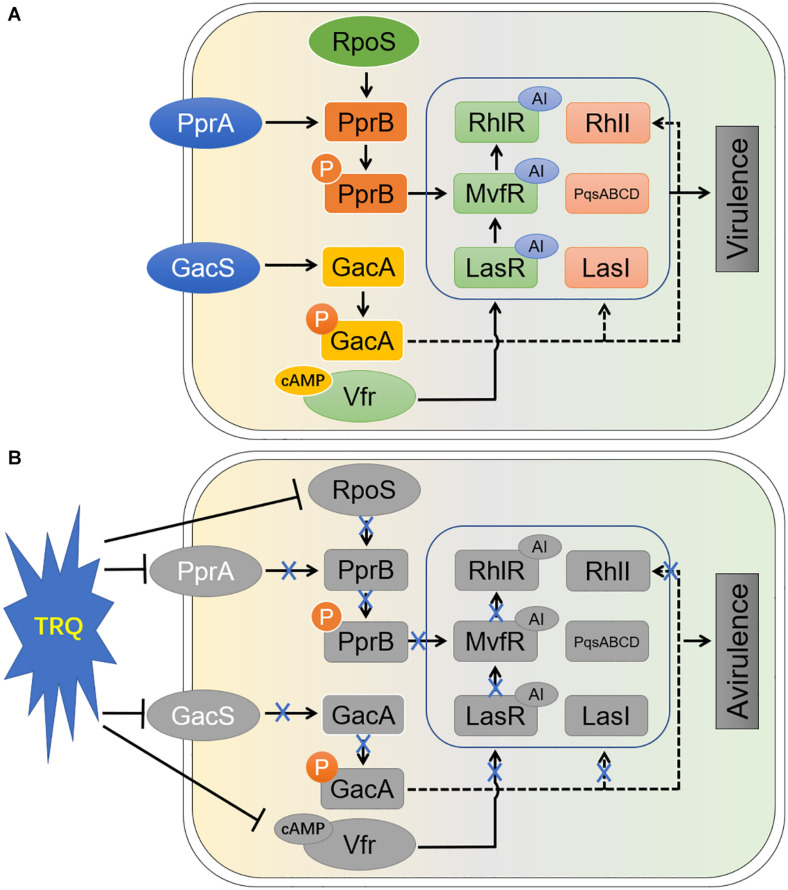
Schematic diagram of the mode of action of TRQ-mediated virulence inhibition in *P. aeruginosa*. Without introduction, the QS systems and their upstream regulators work smoothly **(A)**. In our study **(B)**, we have found that TRQ could efficiently inhibit the transcription of upstream regulators of QS systems in *P. aeruginosa*. Particularly, we found that two TCSs, GacS/GacA and PprA/PprB, were involved in these process. One of the sigma factors, RpoS, was also identified to positively affect the expression of PprA/PprB system (Wang et al., 2019). The expression of c-AMP synthesis was also inhibited by TRQ treatment, suggesting that the downstream Vfr-mediated QS activation via *lasR* was shutdown. In addition, TCS GacS/GacA was found to be involved in the regulation of QS signal synthesis by LasI and RhlI (Reimmann et al., 1997). With the introduction of TRQ, all those processes might be affected and thus the virulent phenotypes diminished (avirulence). AI, autoinducer; P, phosphorylated.

Based on these facts, the complete inhibition of the three hierarchical QS systems in *P. aeruginosa* raises the possibility that TRQ is likely functioning as a multitarget inhibitor of bacterial virulence. Since we have seen that the expression of all three regulator genes (*lasR*, *rhlR* and *mvfR*) were completely repressed by TRQ treatment. One of the QS inhibition mechanisms might be the shutdown of *las* by TRQ and in turn the downregulation of *pqs* and *rhl* systems. The second hypothesis is the inhibition of the *pqs* system by TRQ with the consecutive shutdown of the *rhl* system. In addition to QS inhibition, we have unraveled that two TCSs, GacS/GacA and PprA/PprB, could be inhibited by TRQ treatment, further explaining the reason of QS inhibition and the final attenuation of *P. aeruginosa* virulence. Altogether, we summarized the conclusions from our study in Figure 8.

## Data Availability Statement

The datasets generated for this study can be found in the accession number GSE141753.

## Author Contributions

YW conceived the project and supervised the study. QW, WY, QT, and KC performed the experiments and analyzed the data. GH, LL, and LM performed experiments, provided the reagents, technical assistance and interpretation of data for the project. QW, WY, PC, and YW wrote the draft. All authors added comments and corrections and approved the final version to be published.

## Conflict of Interest

The authors declare that the research was conducted in the absence of any commercial or financial relationships that could be construed as a potential conflict of interest.

## Supplementary Material

Supplementary Table 1 TRQ-treated *P. aeruginosa* regulon in exponential growth phase.

Supplementary Table 2 TRQ-treated *P. aeruginosa* regulon in stationary growth phase.

Supplementary Table 3 Raw expression data of selected genes from TRQ-treated *P. aeruginosa* regulon.

Supplementary Table 4 Main components of three herbal medicines of TRQ (Ru et al., 2014).
